# Isolation and Characterization of a Crude Oil-Tolerant Obligate Halophilic Bacterium from the Great Salt Lake of the United States of America

**DOI:** 10.3390/microorganisms13071568

**Published:** 2025-07-03

**Authors:** Jonathan Oakes, Johurimam Noah Kuddus, Easton Downs, Clark Oakey, Kristina Davis, Laith Mohammad, Kiara Whitely, Carl E. Hjelmen, Ruhul Kuddus

**Affiliations:** Department of Biology, Utah Valley University, Orem, UT 84058, USA; joakes@med.wayne.edu (J.O.); johurimam@gmail.com (J.N.K.); emichaeldowns@gmail.com (E.D.); clarkoakeydds@gmail.com (C.O.); kristina.davis@uvu.edu (K.D.); laithgmohammad@gmail.com (L.M.); kwhitley@uvu.edu (K.W.); carl.hjelmen@uvu.edu (C.E.H.)

**Keywords:** *Salinivibrio costicola*, obligate halophile, biofilm, bioremediation

## Abstract

Most large-scale crude oil spills occur in marine environments. We screened easily propagable/maintainable halophiles to develop agents for the bioremediation of marine spills. A bacterial strain isolated from a polluted region of the Great Salt Lake was characterized and tested for its ability to degrade crude oil. The strain (*Salinivibrio costicola)* is motile, catalase- and lipase-positive, a facultative anaerobe, and an obligate halophile. Its growth optimum and tolerance ranges are: NaCl (5%, 1.25–10%), pH (8, 6–10), and temperature (22 °C, 4–45 °C). Its genome (3,166,267 bp) consists of two circular chromosomes and a plasmid, containing 3197 genes, including some genes potentially relevant to hydrocarbon metabolism. The strain forms a biofilm but is considered nonpathogenic and is sensitive to some common antibiotics. Lytic bacteriophages infecting the strain are rare in the water samples we tested. The strain survived on desiccated agar media at room temperature for a year, grew optimally in complex media containing 0.1–1% crude oil, but failed to reduce total recoverable petroleum hydrocarbons from crude oil. Thus, a recalcitrant halophile may endure crude oil without mineralizing. Due to some of their advantageous attributes, such strains can be considered for genetic manipulation to develop improved agents for bioremediation.

## 1. Introduction

Crude oil spills can happen in any habitat, but most major spills take place in marine environments. Terrestrial pipeline failures and train derailments usually release less than 10,000 barrels of crude oil (bbl) [1], while marine spills releasing as much as 5,000,000 bbl have been reported [2]. Approximately 17,000,000 bbls of crude oil spill into the marine environment each year [3]. The major contributors (in terms of percentage of total crude oil spills) include tanker and ship maintenance (20%), natural seepage from undersea oil veins (8%), tanker collapse (5%), and offshore drilling operations (2%) [3]. Marine oil spills have short-term and long-term effects on marine biota. The short-term effects include scalding injuries resulting from fire, acute toxicity, suffocation, tissue necrosis, reduced oxygenation of water, and blanketing of the bottom mud [4]. The long-term effects depend on the chemical composition of the crude oil and the local physical and chemical factors. The long-term effects include the passage of petrochemicals to the food chain, carcinogenesis, and the displacement of populations [4,5]. The spills and cleanup efforts also affect the local economy [4,5,6].

Cleanup efforts for marine spills encompass the physical removal of oil through skimming, in situ burning, adsorption using various matrices, dissipation with detergents and dispersants, high-pressure hot water washing, chemical stabilization of oil with elastomers, and bioremediation [7,8]. Bioremediation measures, such as phytoremediation, mycoremediation, and prokaryotic remediation, have been successfully utilized to clean up large oil spills [9,10]. While all bioremediation methods can be applied in terrestrial and freshwater environments, prokaryotic remediation is better suited for marine environments.

An ideal bioremediation agent is expected to be a non-fastidious microbe that can be rapidly grown in situ, or propagated, stored, and transported on a large scale with minimal effort, nonpathogenic to plants and animals [11], and relatively immune to viral infections [12]. Genetic endowment to metabolize hydrocarbons, the ability of the agent to survive in diverse environmental conditions, form a biofilm, and secrete biosurfactants are some of the additional essential/desirable traits [13]. Various groups of microbes, including bacteria, archaea, and fungi, can effectively participate in marine bioremediation, and both aerobic and anaerobic microbes may play a role in this process [9]. *Acinetobacter calcoaceticus*, *Yarrowia lipolytica* [14], *Saccharomyces cerevisiae* [15], *Pseudomonas aeruginosa* [16], *Pseudomonas putida* [17], and a mixture of several bacterial species [18], are among the agents reported as successfully aiding in the bioremediation of oil spills. Halophiles capable of degrading crude oil could be ideal for marine bioremediation. Several halophilic bacteria, including the salt-tolerant species of the genera *Alcanivorax*, *Bacillus*, *Gordonia*, *Halomonas*, *Marinobacter*, *Pseudomonas*, *Thalassolituus*, *Rhodococcus*, and *Oleispira* [19,20,21,22], as well as many halophilic archaea with the ability to degrade hydrocarbons and crude oil have been reported [23,24,25,26]. Here, we report the isolation and characterization of a crude-oil-tolerant obligate halophile bacterium capable of growing across a wide range of temperatures and salinities, which can be mass-produced and stored with minimal effort. The bacterium, however, failed to mineralize crude oil, indicating that some recalcitrant microbes may tolerate pollutants without degrading them.

## 2. Materials and Methods

### 2.1. Isolation of the Bacterial Strain

Water and sediment were initially collected from the southeastern region of the Great Salt Lake near Saltair (40°44′49″ N 112°11′17″ W). The samples were transported to the laboratory in an icebox and immediately streaked on a Luria–Bertani (LB) agar plate made with cell-free (ultrafiltered) water collected from the Great Salt Lake. Colonies selected from the plates were transferred to LB broth containing 10% NaCl (LB10) or LB agar plates containing 10% NaCl (LBA10) and incubated at room temperature for 12 to 48 h. Two isolates forming pink and white colonies, respectively, grew most vigorously at room temperature. A pure culture was established from these two samples through repeated streaking on LBA10. A sample from the fifth round of streaking was grown in LB10 and stored at −82 °C for over six months in LB10 containing 30% glycerol. Only the white colony-forming isolate (named as GSL5) survived long-term storage at −82 °C. All subsequent studies were conducted on the strain GSL5.

### 2.2. Preliminary Identification, Microscopy, and Biochemical Tests

Genomic DNA from the isolate was extracted using the cetyltrimethylammonium bromide-NaCl (CTAB-NaCl) precipitation method [27] from 1.5 mL of an overnight culture and served as the template for amplifying a portion of the 16S ribosomal RNA (rDNA) gene with universal primers [28]. Both strands of the amplified DNA fragments were sequenced by an external vendor (Gene Gateway, Hayward, CA, USA). The 16S rDNA sequences were used for a nucleotide BLAST search (https://blast.ncbi.nlm.nih.gov/Blast.cgi, accessed 24 July 2024) to identify the isolate initially. A pure culture grown on LBA10 was utilized for light microscopic examinations and standard microbiological (biochemical and biophysical) tests. All biochemical test media contained 5–10% NaCl. Salt tolerance was assessed using LB broth with added NaCl concentrations ranging from 0% to 24.5%. Since LB contains 0.5% NaCl, the final NaCl concentration varied from 0.5% to 25%. Tolerance to different pH levels was tested in LB10 adjusted to pH 3.0 to 11 by adding 5N NaCl or 5N NaOH. Temperature tolerance was evaluated in LB10 incubated at 4–45 °C. The indicated media inoculated with GSL5 or the control (i.e., sterile LB10) was incubated with moderate shaking (200 rpm) for 24 h; bacterial growth was estimated by measuring A600 of 0.2 mL of culture medium in a 96-well plate (in quadruplet), using a BioTek Synergy I plate reader (Agilent, Santa Clara, CA, USA). Antibiotic resistance and susceptibility were tested on Mueller–Hinton agar plates containing 10% NaCl and incubated at room temperature for 24 h. The resistance/susceptibility was ascertained by measuring the zone of inhibition (ZOI) as described [29].

### 2.3. Genome Analysis

Genomic DNA was extracted from the strain GSL5 using the CTAB-NaCl method [27]. The extracted DNA was cleaned using QIAprep Spin columns (Qiagen, Foster City, CA, USA). DNA was quantified using a NanoDrop spectrometer (Thermo Fisher, Waltham, MA, USA). A sample of 0.2 micrograms of DNA was sequenced using the Rapid Sequencing Kit (SQK-RAD004, version RSE_9046_v1_revAD_14Aug2019, Oxford Nanopore Technologies, Oxford, UK), Flongle flow cells, and the MinION portable DNA sequencer (Oxford Nanopore, Oxford, UK). The software package GUPPY version 6.5.7 (www.search biol.tools, accessed 24 July 2024) was used for base calling and generating FASTQ files. The FASTQ files were assembled into contigs using FLYE version 2.9.1 [30] to generate FASTA files. The FASTA files were utilized for sequence homology analysis using nucleotide BLAST (https://blast.ncbi.nlm.nih.gov/Blast.cgi?PROGRAM=blastn&PAGE_TYPE=BlastSearch&LINK_LOC=blasthome, accessed 24 July 2024) and annotated with Prokka version 1.14.6 [31] through Galaxy [32]. The genome read map was created using CLC Genomics Workbench (CLC Genomics GmbH, Berlin, Germany), and MuMmer4 [33] was employed to compare the genome of GSL5 with related bacteria. The assembled genomic data for strain GSL5 have been submitted to NCBI (PRJNA1226227).

### 2.4. Testing Crude Oil Tolerance of the Strain GSL5

Crude oil was purchased from ONTA Inc. (Toronto, Ontario, Canada). The lot included samples from North America, South America, the Russian Federation, the Gulf States of Asia, and the North Sea. Equal volumes of the crude oil samples from all five regions were pooled together. The mixed sample (0.2 mL) was tested for microbial contamination by growing on 20 mL LB10 and LBA10 (final concentration 1%) before being used to assess the crude oil metabolism and tolerance of the strain GSL5 (no microbial growth was observed in plates streaked with the crude oil sample after a month of incubation at room temperature). Six different growth test media (TM-0 through TM-5), including the essential mineral medium (TM-1), were developed to assess crude oil tolerance and metabolism (Table 1). The essential mineral medium [34] contained 0.1% NH_4_Cl, 0.7% MgCl_2_·6H_2_O, 0.96% MgSO_4_·7H_2_O, 0.05% CaCl_2_·2H_2_O, 0.04% KCl, 0.04% K_2_HPO_4_·3H_2_O, 0.30% NaHCO_3_, 1% NaCl, and 0.1% Na_2_CO_3_ in tap water. The TM0-TM5 media contained 10% NaCl and 1.5% agar; TM-3, TM-4, and TM-5 also contained 1% (*v/v*) crude oil (Table 1). Since oil does not mix with the aqueous medium, Petri dishes (100 mm × 15 mm) were poured with 20 mL of melted agar medium, cooled down to room temperature, and then 0.2 mL of crude oil was spread on the surface of the medium. The plates were inoculated by spreading 0.1 mL of an overnight bacterial LB10 culture (in duplicate). Bacterial growth on the agar plates was approximated by placing the plates on a grid paper (grid size 2.5 mm^2^), counting the number of grids covered by the bacterial colonies (n), then determining the fraction (in %) of the surface area covered by colonies using the formula (n × 2.5 mm^2^ × 100)/total surface area (i.e., 7854 mm^2^ for the 100 mm plate).

### 2.5. Testing Crude Oil Metabolism by the Strain GSL5

A sample of 10 mL of bacterial culture (grown overnight in LB10) was added to 500 mL of LB10 containing 0.1% crude oil in a 2 L Erlenmeyer flask. A control flask contained 500 mL of LB10 containing 0.1% crude oil, but no added bacteria. The flasks were incubated at room temperature with moderate agitation (200 rpm). Bacterial viability was determined every two weeks by plating 0.1 mL of the culture medium from the flasks on LBA10. After 67 days of culture, 250 mL of the sample from each flask was sent to ALS Environmental Group (Salt Lake City, UT, USA) for oil, grease, and total recoverable petroleum hydrocarbons (TRPH) analysis (EPA Test 1664A).

### 2.6. Biofilm Assay

The ability of the strain GSL5 to form biofilm was assayed as previously described [35]. Briefly, the strain GSL5 and a positive control (i.e., *Pseudomonas aeruginosa* strain 27853, obtained from ATCC, Manassas, VA, USA) were grown overnight at 35 °C in LB10 (for GSL5) or LB broth (for *P. aeruginosa* 27853). The overnight culture was diluted 1:100 in the broth medium, and 0.2 mL of the diluted sample was plated in 96-well Costar polystyrene plates (Corning, NY, USA), in quadruples. The plates were incubated for 20 h at 37 °C. Sterile wells containing LB broth served as the negative control. Bacterial growth was estimated by measuring A600 using a Synergy H1 plate reader (Agilent, Santa Clara, CA, USA). The medium was aspirated, and the plates were washed three times with phosphate-buffered saline before being air-dried. Dry methanol (0.2 mL) was added to the wells, and the plates were incubated at room temperature for 15 min. The methanol was then aspirated, and the plates were air-dried. Next, 0.2 mL of a 0.2% crystal violet solution (in water) was added to the plate, and it was incubated at room temperature for 5 min. The stain was then removed, and the wells were washed by gently submerging them in a bucket of water. After the plates were air-dried, 0.16 mL of 33% glacial acetic acid was added to the wells, and the plates were incubated at room temperature for 5 min. Finally, 0.15 mL of the samples was transferred to a new plate, and absorbance (A570) was measured using a BioTek Synergy H1 plate reader (Agilent, Santa Clara, CA, USA).

### 2.7. Susceptibility to Bacteriophage Infection

Since lytic phages can drastically reduce bacterial cell counts, reducing the efficiency of the target biological activities of the cells [36], we tested whether GSL5 is an easy target for lytic phages. We assessed susceptibility to phage infection using a spot assay [37]. Water samples for phages were collected from Wastewater Treatment Facility, Orem, UT, USA (111°41′40.7364″ W); the Great Salt Lake near Saltair (40°44′49″ N 112°11′17″ W), the Pacific Ocean at the Doheny Beach, Dana Point, CA, USA (33°27′39″ N 117°40′41″ W), and the Shoreline Park beach, Mountain View, CA, USA (37.4322° N, 122.0867° W). In addition, mud samples were collected from Salton Sea beach, Imperial County, CA, USA (33.3750° N, 116.0119° W) and suspended in LB10. The water and suspended mud samples were stored on ice and transported to the laboratory. The samples were processed and assayed for phages as described previously [38]. Briefly, the samples were centrifuged at 13,000× *g* to remove any cellular debris and then filtered using a 0.45-micron filter cartridge. Phages were enriched as described [39] with slight modification. Briefly, 1 milliliter of the filtrate was mixed with 1 milliliter of an overnight culture of GSL5 and 8 milliliters of LB10 in a 50-milliliter conical tube. The mixture was incubated at room temperature for 24–48 h with moderate agitation (200 rpm). The bacterial culture was then centrifuged at 13,000× *g* for 3 min, and the supernatant was subsequently passed through a 0.45 μM filter cartridge. The filtrate samples were serially diluted with LB10, and 10 microliters of these samples were spotted on overlay soft agar plates inoculated with GSL5. For a positive control, similarly processed water samples from the Great Salt Lake and Orem City wastewater were spotted on overlay agar inoculated with *Pseudomonas aeruginosa*. The plates were sealed with parafilm, inverted, and incubated at room temperature. Plaque formation was examined visually every 24 h for one week and documented digitally.

## 3. Results

### 3.1. Microscopic, Biochemical, and Biophysical Characteristics

**Figure 1:**
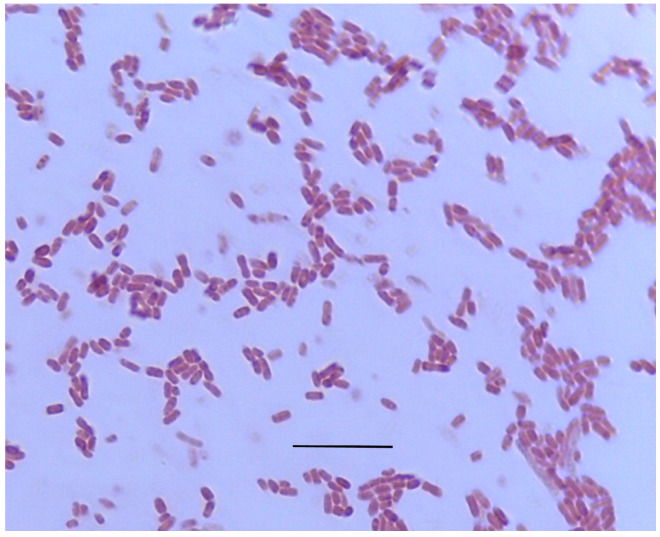
Strain GSL5 habit. Shown is a Gram-stained slide to which log-phase bacteria were fixed. Cells are approximately 1–1.8 μM × 0.4–0.6 μM. Bar is 5 μM.

The 696 bp 16S rDNA gene fragment amplified by universal primers from DNA extracted from the GSL5 strain was >99% identical (query cover 98%, percent identity 99.56–99.71%) to the 16S rDNA of *Salinivibrio costicola* (GenBank Accession No. KY421119.1). Light microscopic examination indicated that the bacterium is a highly motile Gram-negative pleomorphic curved rod (Figure 1). Biochemical tests yielded the following results: Catalase, glucose fermentation, lactose fermentation, lipase, motility, oxidase, and Voges–Proskauer (VP) test were positive; amylase, hydrogen gas production, hydrogen sulfide production, indole test, mannitol fermentation, and methyl red (MR) test were negative; and the bacterium was a facultative anaerobe.

**Figure 2:**
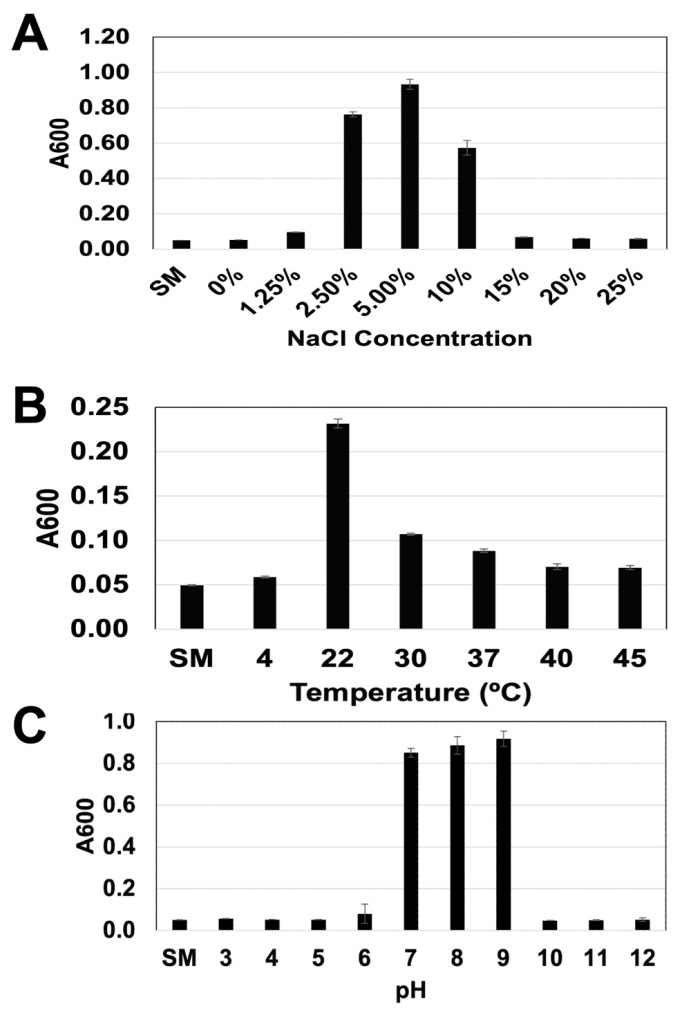
Range and optimum of some physical and environmental factors for growth of GSL5. (**A**) NaCl tolerance. (**B**) Temperature tolerance. (**C**) pH tolerance. Bacterial abundance was estimated by measuring A600. Mean and standard deviation were calculated from four sets of data points.

The salt (NaCl), temperature, and pH tolerance range, as well as the optimum for the growth of GSL5, are shown in Figure 2. The strain GSL5 failed to grow in LB (i.e., 0.5% NaCl) and 15–25% NaCl (Figure 2A). The bacterium grew poorly in 1.25% NaCl and most optimally at 5% NaCl. The bacterium exhibited very poor growth at 4 °C, 40 °C, and 45 °C, but grew optimally at 22 °C (Figure 2B). It failed to grow at pH 3–5 and 10–12, grew poorly at pH 6, but grew optimally at pH 7–9 (Figure 2C). The isolate is resistant to metronidazole (ZOI-0 mm), intermediate/resistant to bacitracin (ZOI-10 mm) and gentamycin (ZOI-14 mm), and sensitive to amoxicillin (ZOI-20 mm), polymyxin B (ZOI-24 mm), sulfamethoxazole-trimethoprim (ZOI-32 mm), doxycycline (ZOI-34 mm), and chloramphenicol (ZOI-35 mm) (Supplemental Data Figure S1).

### 3.2. Phage Infection

Water or mud samples collected from two different points in the Pacific Ocean, the Great Salt Lake, the Salton Sea, and the Orem Wastewater Processing Plant, enriched for phages, produced no plaques on soft agar lawns inoculated with GSL5. In the control experiments, water samples obtained from the Orem Wastewater Processing Plant produced plaques; water samples from the Great Salt Lake produced no plaques, on the overlay agar plate lawns of *Pseudomonas aeruginosa* (Supplemental Data Figure S2).

### 3.3. Ability to Form a Biofilm

**Figure 3:**
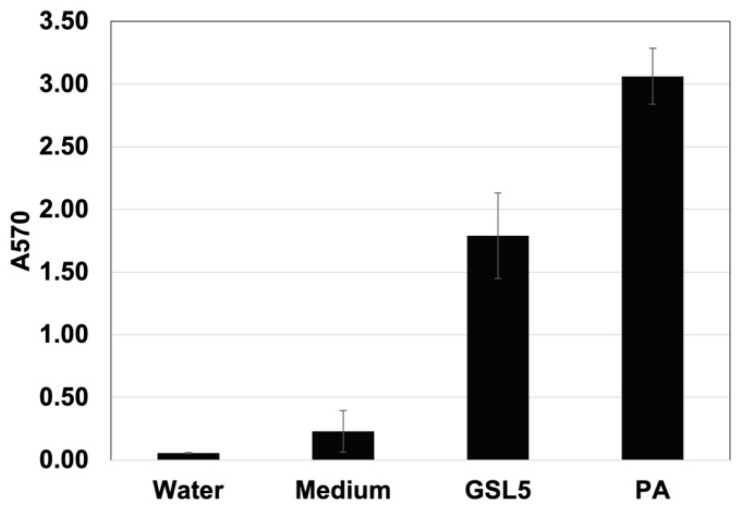
Ability of GSL5 to form biofilms on wells of a polystyrene plate. Mean and standard deviation of four sets of data points for A570 of deposited crystal violet are shown. Water—distilled water lacking any bacterial cells, Medium—LB10 lacking any bacterial cells, GSL5—GSL5 grown in LB10, PA—*Pseudomonas aeruginosa* grown in LB broth.

Biofilm formation (assessed by A570 absorption of deposited crystal violet) by GSL5 is shown in Figure 3. The biofilm formed by GSL5 in 20 h in LB10 on plastic plates was 1.79 ± 0.34 A570 units, which is significantly higher than the negative controls (water—0.06 ± 0.004 A570 units, and cell-free LB10 medium—0.23 ± 0.17 A570 units), but significantly lower than that of *P. aeruginosa* in LB broth medium (3.06 ± 0.22 A570 units).

### 3.4. Crude Oil Tolerance and Metabolism

**Figure 4:**
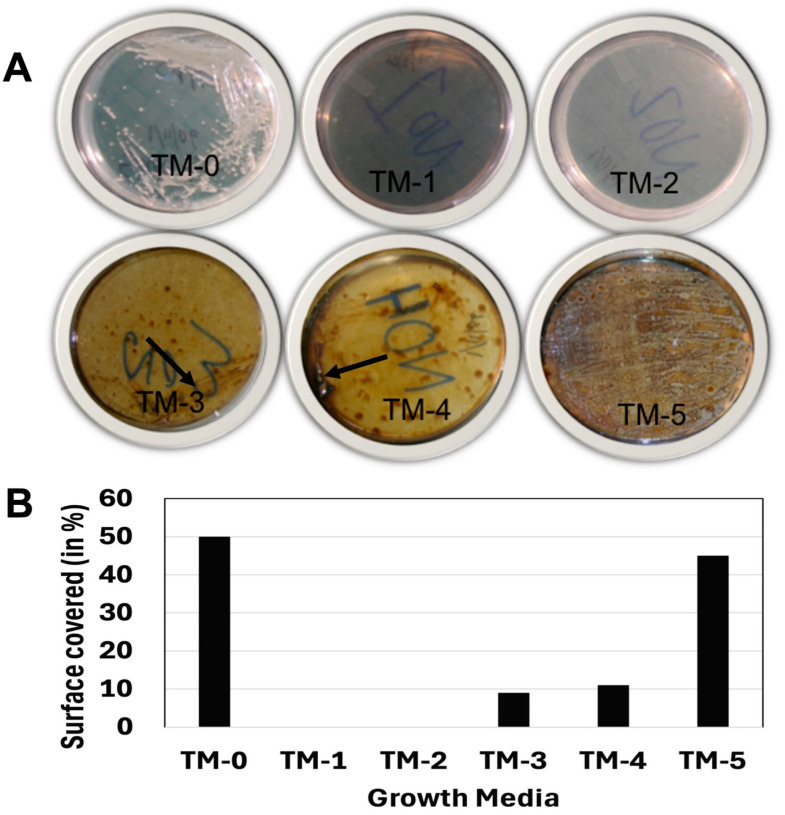
Growth of GSL5 in minimum essential and other growth media. (**A**) Growth on streaked agar plates. All plates were inoculated with equal volumes of bacterial culture. An arrow indicates colonies on TM-3 and TM-4 plates. (**B**) Relative growth of GSL5 on plates, in terms of fraction of area (in %) covered by colonies and lawns. Magnified images of plates, showing the structure of the colonies, are presented in Supplemental Data Figure S3.

Six different media (Table 1) were used to test the metabolic range and crude oil tolerance of GSL5. GSL5 formed a lawn in TM-0 (rich organic medium). The strain was unable to form colonies on TM-1 (essential minerals) or TM-2 (essential minerals containing 1% glucose) (Figure 4A). The strain formed colonies on TM-3 (essential minerals containing 1% crude oil) and TM-4 (essential minerals containing 1% glucose and 1% crude oil) but only in the densely streaked areas (Figure 4A). The strain formed a lawn and many colonies on TM-5 (rich organic medium containing 1% crude oil) but primarily around oil drops. The approximate relative growth in different media compared to TM-0 was 0% in TM-1 and TM-2, about 10% in TM-3 and TM-4, and approximately 90% in TM-5 (Figure 4B).

Since the strain formed colonies on the semisolid media TM-3 (essential minerals containing 1% crude oil) and TM-4 (essential minerals containing 1% glucose and 1% crude oil) but did not grow vigorously yet thrived in TM-5 (rich organic medium containing 1% crude oil), we tested whether the bacterium metabolizes crude oil in the TM-5 broth culture. The bacterium thrived in TM-5 broth containing 0.1% crude oil for over two months, showing no reduction in cell viability. However, the EPA Test 1664A conducted on day 67 showed 21 mg/liter of total recoverable petroleum hydrocarbons (TRPH) in the bacterial culture and a bacteria-free culture medium containing 0.1% crude oil (Supplemental Data Figure S4), indicating that the strain failed to mineralize crude oil.

### 3.5. Genomic Analyses

The assembly of the nucleotide sequences from the genomic DNA library produced three contigs: contig one at 2,619,414 bp, contig two at 549,505 bp, and contig three at 2626 bp, all of which are covalently closed circular DNA molecules. Based on the fold coverages (70-fold for contig 1, 63-fold for contig 2, and 2221-fold for contig 3), GUPPY predicted the genome to consist of one copy each of contigs 1 and 2, and approximately 33 copies of contig 3. Prokka predicted that the genome contains 3197 genes, including 2073 coding DNA sequences (CDS), 28 rRNA genes, 95 tRNA genes, one tmRNA gene, and two repeat regions. The genome contains several genes, including monooxygenases, dioxygenases, alcohol dehydrogenase, aldehyde dehydrogenase, multidrug resistance ABC transporter ATP-binding/permease protein genes, several enzymes involved in surfactant production (including phosphoglucomutases and acyltransferases), and transporters (including sulfate transporters and Zinc/cadmium/lead-transporting P-type ATPase) (Supplemental Data Table S1) involved in bacterial crude oil degradation [40]. The analysis also indicated that the genome lacks genes for several key enzymes involved in bacterial crude oil degradation, including alkane monooxygenase, long-chain alkane hydrolase, and monooxygenase active on aromatic compounds [41].

**Figure 5:**
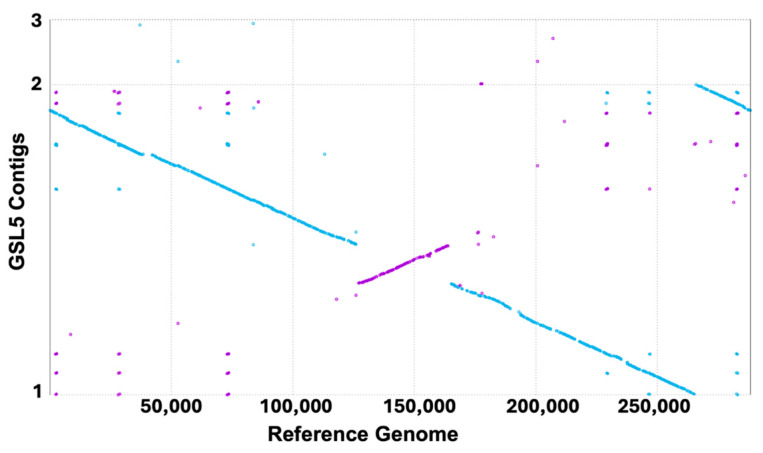
Comparing genomes of strain GSL5 and *Salinivibrio costicola* strain M318. A Mummerplot dot plot comparison of the genome of GSL5 versus the genome of *S. costicola* strain M318 (GenBank GCA_011765565.1) is shown. The purple line centered at 150,000 bp indicates an inversion in the genome of strain GSL5.

A nucleotide BLAST (blastn) confirmed that the genome of GSL5 is closely related to the genome of *Salinivibrio costicola* strain M318 (reference genome GenBank GCF_011765565.1). A Mummerplot generated by comparing the genome of GSL5 with the GenBank reference sequence GCF_011765565.1 using CLC Genomic Workbench indicated an inverted sequence in the genome of GSL5 starting at roughly: (1265872, 925827)–(1641217, 1266049) (Figure 5).

## 4. Discussion

Bioremediation aims at completely mineralizing chemical pollutants. Bioremediation of crude oil is challenging because it contains (or releases upon partial degradation) a multitude of contaminants, including alkanes, alkenes, and aromatic hydrocarbons of various sizes and structures, as well as some heavy metals and nitrogen- and sulfur-containing compounds; some are volatile, some are partially soluble, and some are completely insoluble in water [42]. Whether in situ or a bioreactor, the bioremediation of such a versatile pollutant requires a community of microbes. Fortunately, the list of microbes capable of degrading crude oil is expanding. This list includes genera from bacteria (*Achromobacter*, *Acinetobacter*, *Aeribacillus*, *Aeromonas*, *Alcaligenes*, *Alcanivorax*, *Arthrobacter*, *Bacillus*, *Burkholderia*, *Corynebacterium*, *Enterobacter*, *Escherichia*, *Flavobacterium*, *Gordonia*, *Micrococcus*, *Mycobacterium*, *Nocardia*, *Pseudomonas*, *Psychrobacter*, *Rhodococcus*, *Rhodopseudomonas, Serratia*, *Shewanella*, *Staphylococcus*, and *Vibrio*), archaea (many species of Haloarchaea, including the genera *Haloferax* and *Halobacterium*), algae (*Nitzschia*, *Scenedesmus*, and *Skeletonema*), and fungi (*Aspergillus*, *Fusarium*, *Mucor*, *Penicillium*, *Rhodotorula*, *Sporobolomyces*, *Saccharomyces*, *Candida*, *Yarrowia*, and *Marasmius*) [9,22,23,24,25,26,43,44]. This report explores the potential of bioremediation of *Salinivibrio costicola*. Although *S. costicola* has been reported to have the ability to degrade total organic carbon (TOC) pollutants in wastewater [45], to our knowledge, this report is the first to investigate its suitability as a bioremediation agent and its ability to degrade crude oil.

The goal of this study was to isolate a non-fastidious salt-tolerant bacterium that can be manipulated to develop a strain for bioremediation of marine crude oil spills. We chose to isolate the strain from the south arm of the Great Salt Lake, which experiences significant fluctuations in salinity and water temperature due to the seasonal inflow of freshwater from ice-fed rivers (i.e., Bear, Jordan, Ogden, and Weber rivers), as well as droughts. The lake is experiencing rapid decline due to global climate change. The northern arm of the lake has turned hypersaline (salinity 26–30%) and lost biodiversity due to its separation from the south arm by the railroad causeway [46]. The south arm of the lake is in relatively better condition; however, it is contaminated with numerous industrial pollutants, including arsenic, cadmium, lead, copper, and mercury [47,48]. We also observed that the bacterium is quite resilient, able to recover viable cells after the strain grown on LBA10 plates was stored at room temperature for 15 months to allow the plates to desiccate (Supplemental Data Figure S5). We hypothesized that a microbe flourishing in such harsh conditions could aid in developing effective agents for bioremediation.

The microscopic, biochemical, biophysical, and genomic characteristics of the isolate indicate that our isolate (GSL5) is a strain of *Salinivibrio costicola* (former name *Vibrio costicola*). Like other described strains of *S. costicola* [49], GSL5 is a Gram-negative pleomorphic curved flagellated rod (~1.5 × 0.5 µm). The strain GSL5 is catalase, glucose fermentation, lactose fermentation, lipase, motility, oxidase, and Voges–Proskauer (VP) test positive; amylase, hydrogen gas production, hydrogen sulfide production, indole test, mannitol fermentation, and methyl red (MR) test negative; and a facultative anaerobe. The reported strains of *S. costicola* exhibit metabolically diverse characteristics, including the ability to grow in the absence of NaCl and oxygen by fermenting glucose, mannitol, and other organic compounds. Additionally, some strains of *S. costicola* are amylase- and lipase-negative [49,50]. A detailed biochemical characterization of the strain was beyond the scope of this project. However, we investigated properties that could make the strain suitable for bioremediation of marine environments. GSL5 grows well at NaCl concentrations between 1.25% and 10% (most optimally at 5% NaCl), which falls within the salinity range of seawater and estuarine water [51]. The pH tolerance of GSL5 is narrow (7–9), but it remains within the typical pH range of seawater (8–8.2) [51]. GSL5 can grow across a wide temperature range of 4–45 °C, with optimal growth at room temperature; the temperature range of seawater is −2 to 30 °C [51].

Biofilms help bacteria survive in hostile environments, including toxic conditions [52]. The strain GSL5 forms biofilms efficiently on plastic surfaces, demonstrating a comparable efficiency to known biofilm producers such as *Pseudomonas aeruginosa*. This strain produced smooth, mucoid colonies on LBA10 plates and LBA10 plates containing 1% crude oil (Figure 4), indicating the secretion of an extracellular matrix. Biofilm formation is a virulence factor for pathogenic bacteria [53]. GSL5 is resistant to several antibiotics, including metronidazole and gentamicin, but is sensitive to some common antibiotics such as amoxicillin and chloramphenicol. The biofilm formation and antibiotic resistance of GSL5 raise concerns. However, LPSN (http://www.bacterio.net, accessed on 25 May 2025) categorized *S. costicola* in Risk Group 1 (low risk, no substantial evidence of pathogenicity to humans) [54], and we found no reports of its association with infections in any saltwater organisms.

Infection by lytic phages can drastically reduce the target biological activity of industrially important bacterial strains [36]. Our limited effort (five samples from different locations, enriched by co-culturing with the host bacterium) to test the susceptibility of GSL5 to phage infection returned negative results. Our findings do not prove the immunity of GSL5 to lytic phage infections but may indicate that phages capable of infecting GSL5 are relatively rare in the habitats we investigated. A recent study demonstrated that phage infection may enhance the rate of bioremediation by some bacterial species, likely due to the phage-driven microbial loop effect [55].

Although GSL5 has some attributes of a good bioremediation agent, it is either incapable of or highly inefficient in metabolizing and mineralizing crude oil. Since the bacterium formed colonies in media containing essential minerals along with crude oil but was unable to form colonies in the medium containing only essential minerals (Figure 4), we initially hypothesized that the bacterium degrades or metabolizes crude oil very slowly. However, the EPA Test 1664A indicated that GSL5 was unable to reduce the TRPH from crude oil samples even after growing in it for two months. The EPA Test 1664A estimates total recoverable petroleum hydrocarbons (TRPH) in a crude oil sample; it does not account for changes in the sample’s chemical composition. Therefore, our test cannot reveal whether GSL5 converted some components of crude oil without reducing the total amount of hydrocarbons in the sample. We plan to use analytical tests, including gas chromatography [56], to evaluate any potential changes in the composition of crude oil exposed to GSL5 or genetically improved GSL5 strains in the future.

A comparative genomics study to identify the genetic basis of bacterial degradation of hydrocarbons and survival in oil-contaminated environments [40] identified dozens of genes directly or indirectly involved in crude oil metabolism. Our preliminary analyses identified several related genes, including those for alcohol dehydrogenases, aldehyde dehydrogenases, phosphoglucomutases, acyltransferases, sulfate transporters, and heavy metal transporters, in the genome of GSL5 (Supplementary Data Table S1). Further genomic and biochemical analyses are necessary to evaluate the relevance of the finding. Our studies also found that several key genes involved in hydrocarbon metabolism, including alkane monooxygenases, long-chain alkane hydrolases, and monooxygenases active on aromatic compounds [40,41], are absent from the genome of GSL5.

Whether GSL5 represents a new subspecies of *S. costicola* was not a goal of this investigation. However, we sequenced the 16S rDNA as well as the whole genome of the isolate. The 16S rDNA of GSL5 is 93.7% identical to the 16S rDNA of *S. costicola* strain M318 (NCBI Accession No. NZ_CP050266.1), but 99.6% identical to the 16S rDNA of *S. costicola* strain ATCC 33508 (NCBI Ref Seq: NR_027590.1). Our blastn analyses indicate that the genome of the strain is highly similar but not identical to that of *S. costicola* strain 318 (NCBI RefSeq GCF_011765565.1). There are several differences between the two genomes. For example, the GSL5 genome consists of three chromosomes of sizes 2619 kbp, 550 kbp, and 2.6 kbp (totaling 3172 kbp, containing 3197 genes). In contrast, the genome of *S. costicola* strain 318 is composed of four chromosomes of sizes 2834 kbp, 617 kbp, 74.3 kbp, and 30.1 kbp (totaling 3556 kbp, containing 3343 genes), indicating that the genome of GSL5 is 11% shorter in size and contains about 4% fewer genes than *S. costicola* strain 318. This is not unusual, as different strains of *E. coli* can exhibit genome size and gene content differences of as much as 20% [57]. Moreover, the Mummerplot (Figure 5) indicates an inversion in the genome of GSL5 compared to the *S. costicola* strain 318. Thus, the two strains are different in several important ways. We plan to investigate the phylogenetic relations of GSL5 with other known strains of *S. costicola* in the future.

Remarkable progress has been made in crude oil bioremediation using bacteria [11,12,13,14,15,19,20,21,22,58,59], and archaea [23,24,25,26,60,61]. Kits (such as Ultra-Archaea^®^, Ultratech, Jacksonville, FL, USA) containing assorted archaea for the bioremediation of hydrocarbons in soil and freshwater are now commercially available. Further progress, such as creating genetically modified (GM) microbes for the bioremediation of chemical pollutants, particularly forever petrochemicals, has been an active research area since the early 2000s. This approach can be beneficial in creating agents for marine bioremediation since some highly effective bioremediation agents are terrestrial or freshwater mesophiles. GM bacteria are typically constructed by transferring manipulated naturally occurring or synthetic plasmid vectors [62,63]. However, transposon-assisted gene transfer is also effective [64] and safer because, unlike plasmid vectors, transposon vectors are less active in random horizontal gene transfer [65]. We plan to create genetically modified (GM) strains of GSL5 to test their ability to degrade crude oil. Like *Salinivibrio* sp., many effective bioremediation agents (including the genera *Alcaligenes*, *Acinetobacter*, *Pseudomonas*, and *Shewanella*) are proteobacteria. Therefore, transferring genes from such genera to *Salinivibrio* sp. should be less cumbersome.

Crude oil contamination is an escalating environmental issue affecting terrestrial, freshwater, and marine ecosystems, and its mitigation necessitates an integrated approach [66]. Bioremediation is one of the many tactics within that comprehensive approach. Since pollution alters microbial communities, the modified community attempts to thrive and decontaminate the pollutants [67]. Although microbes in natural environments compete for nutrients, they also cross-feed, as some species utilize the wastes of other species as energy sources [68]. Consortia of natural and genetically modified microbes may help the remaining native community, improve bioremediation, and restore the environment [69,70]. We envision genetically modified GSL5 strains as part of the reconstructed microbial community for restoring polluted marine environments.

## Figures and Tables

**Table 1:** Test media used for crude oil tolerance/metabolism of strain GSL5. All media contained 10% NaCl and 1.5% agar.

Name of the Medium	Description
TM-0	LB broth (LB10)
TM-1	Essential minerals [34]
TM-2	Essential minerals and 1% glucose
TM-3	Essential minerals and 1% crude oil
TM-4	Essential minerals, 1% glucose, and 1% crude oil
TM-5	LB broth and 1% crude oil

## Data Availability

The genome sequence data for the strain GSL5 have been submitted to NCBI (PRJNA1226227).

## Supplementary Materials

The following supporting information can be downloaded at: https://www.mdpi.com/article/10.3390/microorganisms13071568/s1, Table S1. Some genes of GSL5 that may be involved in bacterial biodegradation of crude oil (See References [40,41]). Figure S1: Sensitivity and resistance to certain antibiotics of strain GSL5. Left plate was inoculated with strain GSL5, while right plate was inoculated with *Escherichia coli* (ATCC 25922). Antibiotic discs: 1—metronidazole, 2—gentamycin, 3—bacitracin, 4—amoxicillin, 5—doxycycline, 6—sulfamethoxazole-trimethoprim, 7—chloramphenicol, 8—vancomycin, 9—polymyxin B. Figure S2: Plaque formation on GSL5 and *P. aeruginosa* overlay agar plates by phage-enriched water samples. A—Doheny Beach water sample on GSL5 plate; B—Shore Park water sample on GSL5 plate; C—Salton Sea water sample on GSL5 plate; D—Great Salt Lake water sample on GSL5 plate; E—Orem Westwater Plant water sample on GSL5 plate; F—Great Salt Lake water sample on *P. aeruginosa* plate; G—Orem Westwater Plant water sample on *P. aeruginosa* plate. Media used for plates A through E—LBA10, containing 1.0 mM MgSO_4_, and for plates F and G—trypticase soy agar, containing 1.0 mM MgSO_4_. Dilutions: 0—undiluted, 10, 100, 1k, and 10k, 10M—a hundred, a thousand, ten thousand, or 10 million-fold diluted. Med only—phage-free diluent medium. Figure S3: Growth of strain GSL5 on experimental media. Plates made from media TM-1, TM-2, TM-3, and TM-4 magnified to illustrate bacterial growth. There was no bacterial growth on TM-1 and TM-2, while some growth on TM-3 and TM-4. Figure S4: Lack of reduction of total petroleum hydrocarbons by strain GSL5. Report from ALS Environmental indicates that total petroleum hydrocarbons remained unchanged after cultivating strain GSL5 in LB10 with 0.1% crude oil for 67 days, compared to control (same medium lacking any bacterial cells). Figure S5: Resiliency of strain GSL5. Strain was grown on LBA10 plates on 31 May 2022. Left plate was sealed with parafilm and stored at room temperature. On 21 September 2023, the plate was washed with 100 microliters of sterile water. Samples of sterile water and rehydrated LBA10 plate were streaked on fresh LBA10 plates on 21 September 2023 and incubated at room temperature for 48 h. No colony grew on the middle plate streaked with sterile water, but many colonies grew on the right plate streaked with the rehydrated sample.

## Abbreviations

ATCC	American Type Culture Collection, Manassas, Virginia
bbl	barrel
EPA	Environmental Protection Agency
GM	genetically modified
GSL5	Great Salt Lake Strain 5 of *Salinivibrio costicola*
LB10	Luria–Bertini broth containing 10% NaCl
LBA10	Luria–Bertini broth containing 10% NaCl and 1.5% agar
NCBI	National Center for Biotechnology Information
rDNA	ribosomal DNA
TM	testing medium
TRPH	total recoverable petroleum hydrocarbons
ZOI	zone of inhibition
