# Supplementary material for: Isolation and Characterization of a Crude Oil-Tolerant Obligate Halophilic Bacterium from the Great Salt Lake of the United States of America
Source: Microorganisms. 2025 Jul 3;13(7):1568. doi: 10.3390/microorganisms13071568 (PMC12299700; doi:10.3390/microorganisms13071568)

**Supplementary Materials:**

**Supplemental Data Table 1.** Some genes of GSL5 that may be involved in the bacterial biodegradation of crude oil [See References 40 and 41].

| Gene | Abbreviation | Possible functions related to bioremediation | Genome location (Chromosomes 1 and 2) |
|---|---|---|---|
| Ammonia monooxygenase | *petC* | Nitrification | 1: 2037308-2038045 |
| Alcohol dehydrogenase | *adhB* | Hydrocarbon degradation | 2: 203951-205099 |
| Alcohol dehydrogenase | *yqhD* | Hydrocarbon degradation | 2: 487931-489088 |
| Aldehyde dehydrogenase | *betB* | Hydrocarbon degradation | 1: 1536289-1537749 |
| Coniferyl aldehyde dehydrogenase | *calB* | Degradation of aromatic aldehydes | 2: 217013-218449 |
| Long-chain-aldehyde dehydrogenase | *ald1* | Degradation of long chain aldehydes | 1: 59735-61243 |
| Homogentisate dioxygenase | *hmgA* | Catabolism of aromatic compounds | 1: 2613597-2614463 |
| Quercetin dioxygenase | *yhhW* | Flavonoid degradation | 2: 231406-232101 |
| Multidrug resistance ABC transporter ATP-binding/permease protein | *bmrA* | Resistance to metal ions, hydrocarbon degradation | 2:238588-240399 |
| Phosphoglucomutase | *pgm* | Surfactant production | 1:1484945-1486591 |
| Phosphate acyltransferase | *plsX* | Surfactant production | 1:791444-7924466 |
| Sulfate transporter | *cysZ* | Sulfate reduction | 1:1648218-1658976 |
| Sulfate transport system permease protein | *cysT* | Sulfate reduction | 2:468540-469406 |
| Zinc/cadmium/lead-transporting P-type ATPase | *zntA* | Heavy metal decontamination | 1: 1296504-1298762 |
| Polyphenol oxidase | *yfiH* | Degradation of phenolic compounds | 1:2113315-2114037 |

**Supplementary Data Fig. 1.** Sensitivity and resistance to certain antibiotics of the strain GSL5. The plate on the left was inoculated with the strain GSL5, while the plate on the right was inoculated with *Escherichia coli* (ATCC 25922). Antibiotic discs: 1 - metronidazole, 2 - gentamycin, 3 - bacitracin, 4 - amoxicillin, 5 - doxycycline, 6 - sulfamethoxazole-trimethoprim, 7 - chloramphenicol, 8 - vancomycin, 9 - polymyxin B.

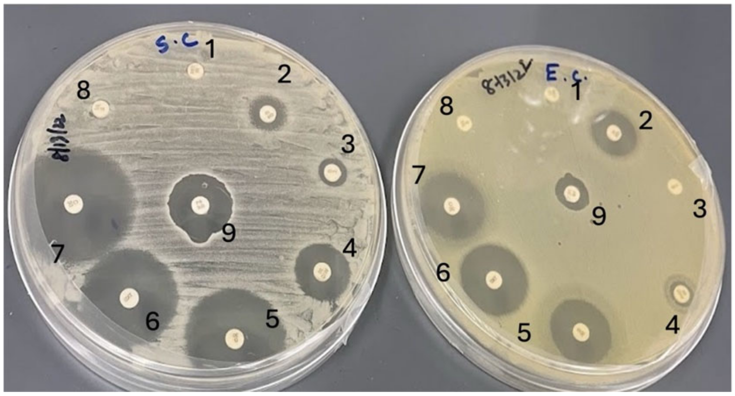

**Supplementary Data Fig. 2.** Plaque formation on GSL5 and P. aeruginosa overlay agar plates by phage-enriched water samples. **(**A) Doheny Beach water sample on GSL5 plate. (B) Shore Park water sample on GSL5 plate. (C) Salton Sea water sample on GSL5 plate. (D) The Great Salt Lake water sample on GSL5 plate. (E) Orem Westwater Plant water sample on GSL5 plate. (F) The Great Salt Lake water sample on *P. aeruginosa* plate. (G) Orem Westwater Plant water sample on *P. aeruginosa* plate. The media used were plates A through E- LBA10, containing 1.0 mM $MgSO_4$, and plates F and G- trypticase soy agar, containing 1.0 mM $MgSO_4$. Dilutions: 0- undiluted, 10, 100, 1k, and 10k, 10M- a hundred, a thousand, ten thousand, or 10 million-fold diluted. Med only- phage-free diluent medium.

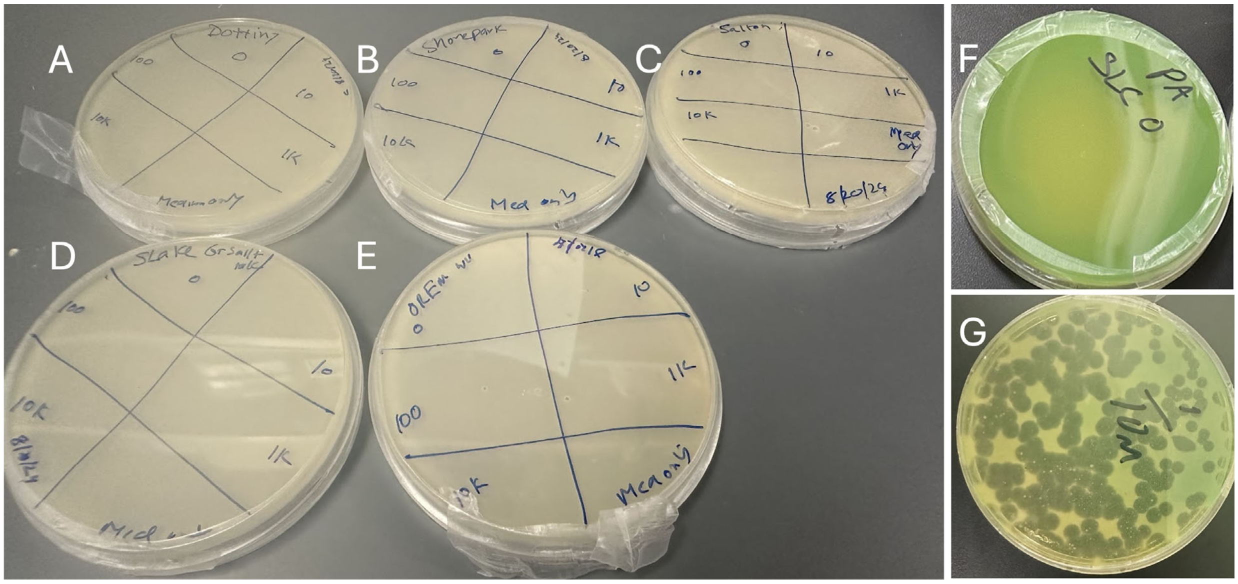

**Supplemental Data Fig. 3.** Growth of strain GSL5 on experimental media. Plates made from the media TM-1, TM-2, TM-3, and TM-4 are magnified to illustrate bacterial growth. There was no bacterial growth on TM-1 and TM-2, while some growth on TM-3 and TM-4.

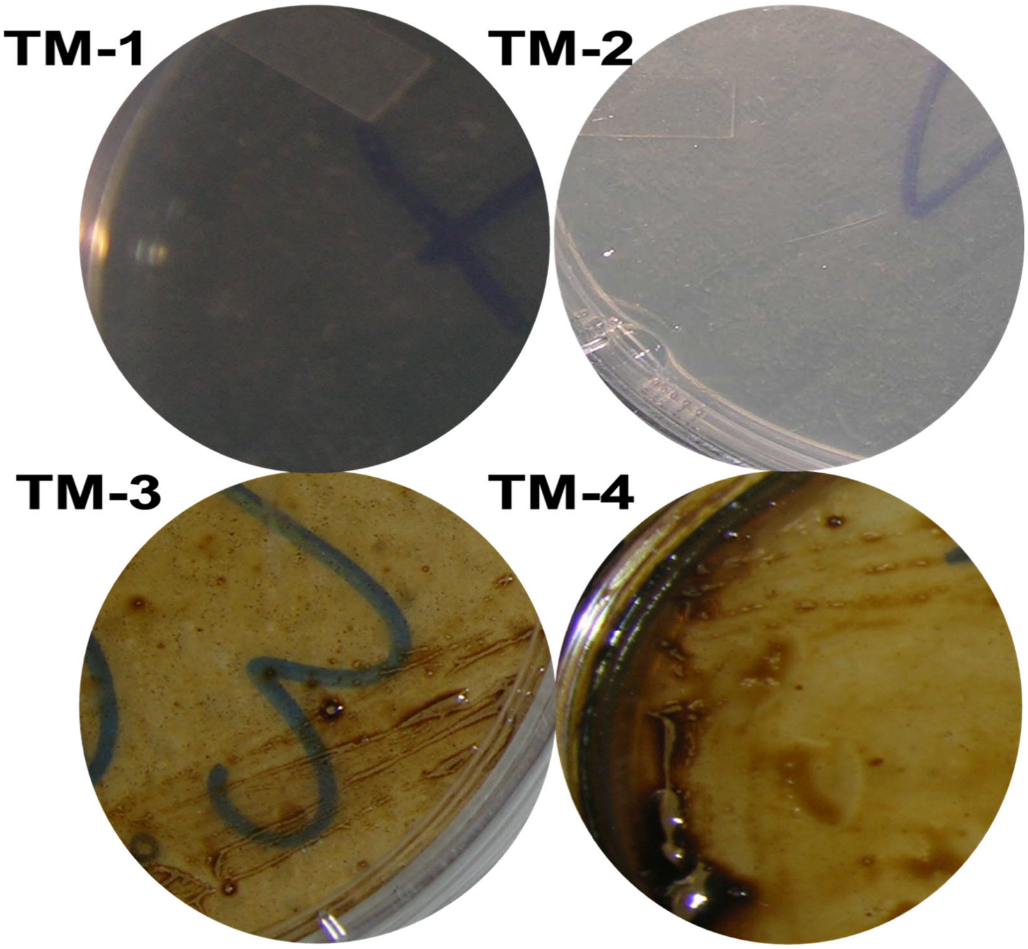

**Supplementary Data Fig. 4.** Lack of reduction of total petroleum hydrocarbons by strain GSL5. The report from ALS Environmental indicates that total petroleum hydrocarbons remained unchanged after cultivating strain GSL5 in LB10 with 0.1% crude oil for 67 days, compared to the control (the same medium lacking any bacterial cells).

ALS Environmental

**1502181**

This report consists of 3 water samples received by ALS on 02/11/15.

**Oil and Grease:**
The samples were prepared and analyzed according to EPA Method 1664A procedures utilizing the current revision of SOP 671.

All acceptance criteria were met.

The data contained in the following report have been reviewed and approved by the personnel listed below. In addition, ALS certifies that the analyses reported herein are true, complete and correct within the limits of the methods employed.

Final Data Reviewer — Date: 2/20/15

**ALS Environmental -- FC** **SAMPLE SUMMARY REPORT**

**Client:** ALS Environmental — **Date:** *20-Feb-15*
**Project:** 1504137 Sal Bacteria — **Work Order:** 1502181
**Sample ID:** 1-Bacteria — **Lab ID:** 1502181-1
**Legal Location:** — **Matrix:** WATER
**Collection Date:** 2/9/2015 13:00 — **Percent Moisture:**

| Analyses | Result | Qual | Report Limit | Units | Dilution Factor | Date Analyzed |
|---|---|---|---|---|---|---|
| **Hexane Extractable Material--Gravimetric** | | | EPA1664 | | Prep Date: **2/17/2015** | PrepBy: **TLB** |
| TOTAL PETROLEUM HYDROCARBONS | 130 | | 21 | MG/L | 1 | 2/20/2015 |

**Client:** ALS Environmental — **Date:** *20-Feb-15*
**Project:** 1504137 Sal Bacteria — **Work Order:** 1502181
**Sample ID:** 3-Neg Cont — **Lab ID:** 1502181-3
**Legal Location:** — **Matrix:** WATER
**Collection Date:** 2/9/2015 13:00 — **Percent Moisture:**

| Analyses | Result | Qual | Report Limit | Units | Dilution Factor | Date Analyzed |
|---|---|---|---|---|---|---|
| **Hexane Extractable Material--Gravimetric** | | | EPA1664 | | Prep Date: **2/17/2015** | PrepBy: **TLB** |
| TOTAL PETROLEUM HYDROCARBONS | 120 | | 21 | MG/L | 1 | 2/20/2015 |

ADDRESS 225 Commerce Drive, Fort Collins Colorado 80524 USA | PHONE +1 970 490 1511 | FAX +1 970 490 1522
ALS GROUP USA, CORP. Part of the ALS Group An ALS Limited Company

1 of 10

**Supplemental Data Fig. 5.** Resiliency of the strain GSL5. The strain was grown on LBA10 plates on 31 May 2022. The plate (on the left) was sealed with parafilm and stored at room temperature. On 21 September 2023, the plate was washed with 100 microliters of sterile water. Samples of sterile water and the rehydrated LBA10 plate were streaked on fresh LBA10 plates on 21 September 2023 and incubated at room temperature for 48 hours. No colony grew on the plate (in the middle) streaked with sterile water, but many colonies grew on the plate (on the right) streaked with the rehydrated sample.

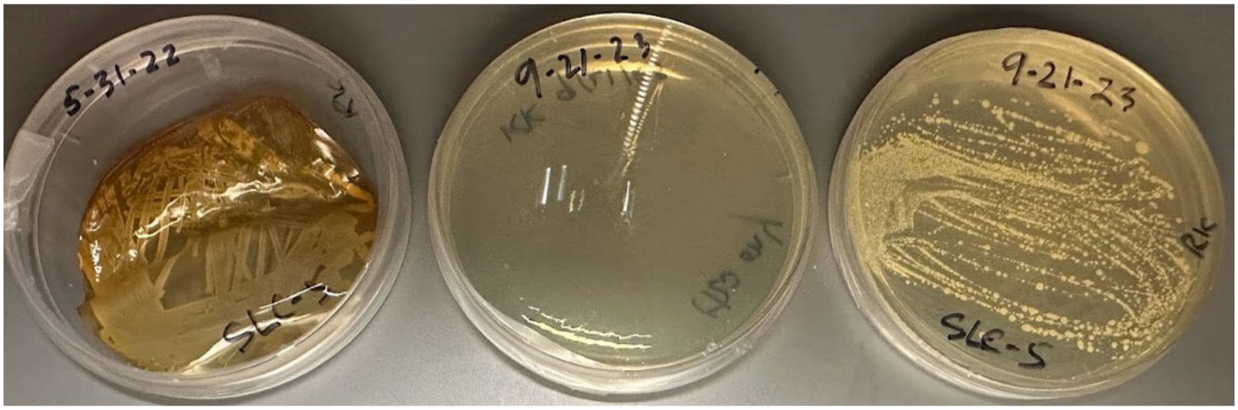

Supplement: Supplementary file 1 [file microorganisms-13-01568-s001.zip › microorganisms-3645373-supplementary.pdf]
